# Do body colour and sociability impact scototaxis response of fish?

**DOI:** 10.1038/s41598-024-67473-0

**Published:** 2024-07-19

**Authors:** Alessandra Pecunioso, Elena Aleotti, Christian Agrillo

**Affiliations:** 1 Department of General Psychology, University of Padova, Padova, Italy; 2 Padua Neuroscience Center, University of Padova, Padova, Italy

**Keywords:** Scototaxis response, *Corydoras aeneus albinus*, *Paracheirodon axelrodi*, *Kryptopterus bicirrhis*, *Hyphessobrycon megalopterus*, Animal behaviour, Neuroscience

## Abstract

Scototaxis test is an anxiety-like test used by behavioural neuroscientists consisting in the assessment of dark/light preference of laboratory animals. This test has been widely used in fish. Most of the species have been shown to express a preference for the dark environment. However, the majority of the investigated species has a dark body colour, thus making a clear contrast with a white/bright background. Also, while in nature fish tend to be highly social, studies in the scototaxis literature tested single fish. Yet, individual vs. group behaviour might interact with scototaxis response. In experiment 1, we assessed the individual response to test the hypothesis that the different colours of the body might modulate the dark/light preference. We found that species with a dark body colour (*Hyphessobrycon megalopterus*) and a largely transparent body colour (*Kryptopterus bicirrhis*) strongly preferred the darker environment. Instead, the preference for darkness of a species with a luminescent part of the body (*Paracheirodon axelrodi*) was less pronounced. Lastly, a species with a white body colour (*Corydoras albini*) did not prefer either a bright or a dark sector. In experiment 2, we explored the behaviour of these species when inserted in shoals of 20 individuals in the experimental apparatus. While *H. megalopterus* and *K. bichirrhis* confirmed their robust preference for darker environments, the other two species changed their preference. Taken together, these results suggest that scototaxis response is context-dependent, as it appears to be modulated by the body colour and the presence/absence of other conspecifics in the surrounding.

## Introduction

When exploring novel environments, animals adopt different strategies to increase survival. Hiding in dark areas (or avoiding brightly lit areas; also known as ‘scototaxis response’) is often adopted by some mammals and amphibians and is particularly investigated in fish species^1–7^. Indeed, the observation of this behaviour is currently used by neuroscientists as a valid low-cost tool for assessing anxiety in laboratory fish (for a review see^8^). Exposing a fish to a half-bright and half-dark environment is supposed to lead to a conflict situation in which the subjects have to trade off their natural tendency to explore the environment and the tendency to hide in a darker area in the presence of a potentially dangerous context^5,8^.

Swimming in a dark environment (i.e., in a cave or the shadows) permits fish to hide from potential predators as they reflect less light, and vision is one of the most important sensory modalities for many fish species. However, we may hypothesize that different species might also modulate this preference for darker environments depending on the colour of their body. For instance, a fish with a transparent or white body might also hide in a bright background, as it might permit to camouflage themselves. Also, bioluminescent species or species with iridescent parts on the body might find more difficult than other fish to hide properly in a dark environment because of the luminance contrast of the iridescent part of the body and, hence, they might be spotted by predators. As far as we are aware, the existing literature on scototaxis is confined to species with a body colour that creates a clear contrast with a white background, and lack evidence about how light and transparent species cope with a light/dark background^9^.

In addition, even though social regimes for fixed social roles are largely absent in fish and social grouping is by majority flexibly exhibited, most of the investigated species in this field live in groups. However, the scototaxis measures are commonly recorded in laboratories with single individuals in the experimental apparatus and, thus, they suffer poor ecological validity. In nature, most of the light–dark responses (e.g., entering into a dark cave) is likely to occur in a social environment and we cannot exclude that the scototaxis response may be modulated by the presence of other companions.

This aligns with the approach of modern cognitive ecologists, an alternative approach of the strictly behaviorist/adaptationist one, in which the decisions of individuals are no longer described as a mere weighing of external stimuli but also as a product of ecological needs that embrace social dynamics too^10^. For instance, the tendency to rest in a darker area might be smaller when animals explore a novel environment in groups/shoals, as animals might benefit from hiding in a group of conspecifics to reduce the chance of being spotted by predators (for confusion effect, many-eye effect, and dilution effect, see^11^). As far as we are aware, this hypothesis has been tested only once with zebrafish, a species whose body colour strongly contrasts with bright backgrounds. Mansur and colleagues^12^ found that the spontaneous preference of zebrafish for darker environments is reduced when shoaling in groups of 8 individuals rather than 4, suggesting that shoaling behaviour might decrease stress and anxiety.

In the present study, we conducted two experiments. In experiment 1, we tested the hypothesis according to which light–dark preference is mediated by the body colour/contrast with the background. In details, we presented four different conditions: (a) Bright body colour/high contrast with dark background/low contrast with white background, (b) Dark body colour/low contrast with dark background/high contrast with white background, (c) Transparent body colour/low contrast with dark background/low contrast with white background, and (d) Iridescent stripe in the body/medium contrast with the dark background/medium contrast with the white background. For the sake of summary, we will generally label our independent variable as ‘body colour’ throughout the manuscript.

**Figure 1:**
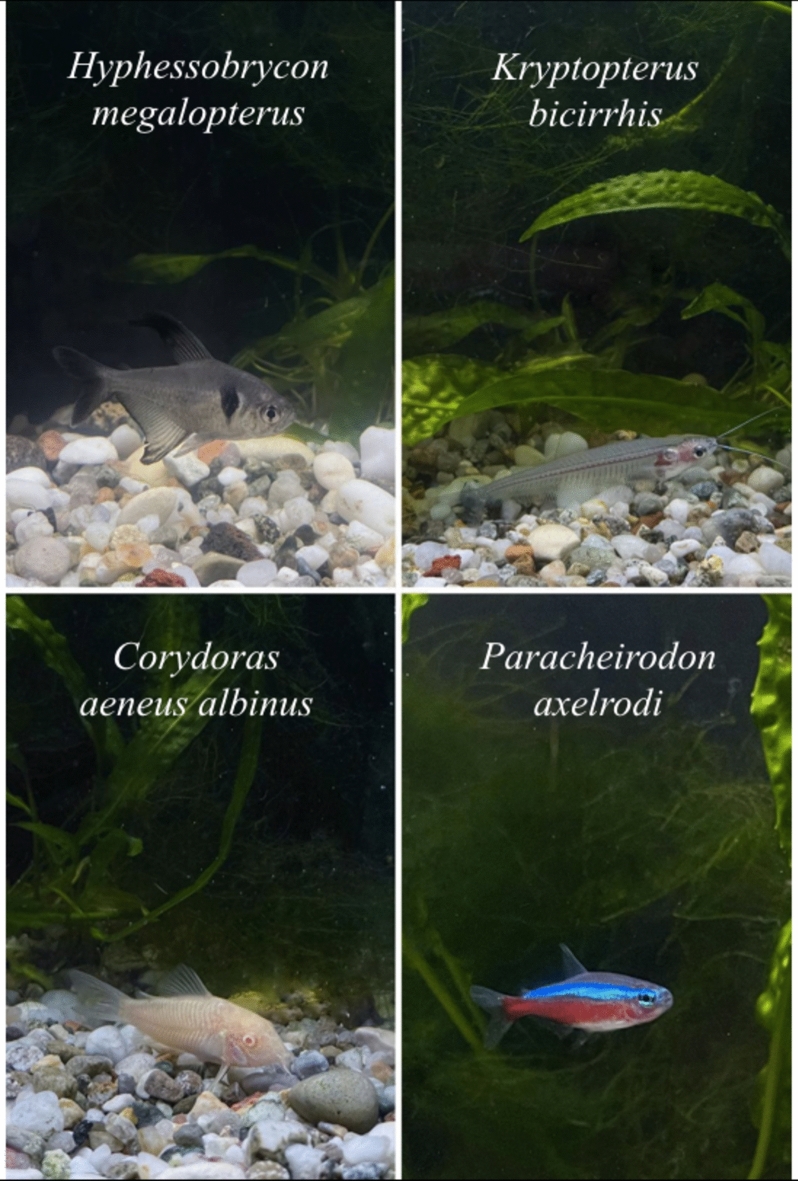
Species under investigation. The four species under investigation: A species with a dark body colour (*H. megalopterus*), a species with a largely transparent body (*K. bicirrhis*), a species with a white body colour (*C. aeneus albinus*) and a species with an iridescent part of the body (*P. axelrodi*).

We tested four different fish species with different body colours (Fig. 1): a species with a white body (*Corydoras aeneus albinus*), a species with a largely transparent body (*Kryptopterus bicirrhis*) and a species with an iridescent part of the body (*Paracheirodon axelrodi*). As a control with the existing literature, we also tested a species with a dark body colour (*Hyphessobrycon megalopterus*). In the case of *Hyphessobrycon megalopterus*, we expected a clear preference for the darker environment, as predicted by the literature on fish^5–7^. For the other species, we still expected a preference for the darker area because no/a few amount of light is reflected by their body, but we predicted it to be less marked than observed in fish with dark body (like in *Hyphessobrycon megalopterus*). In *Corydoras aeneus albinus* and *Kryptopterus bicirrhis*, the lighter/transparent colour of their body may also partially camouflage them with the white floor. Hence, the preference of dark and bright areas might not be significantly pronounced. Finally, we also expected that *Paracheirodon axelrodi* would stay in the darker area more than in the brighter one. Yet, the iridescent blue-green lateral stripe along both sides of the body may make visible individuals also in the darkness although less compared to lightness (at least to human observers), thus reducing the needs to find a darker environment compared to *Hyphessobrycon megalopterus*.

In experiment 2, we explored the possibility that the scototaxis response is different when fish are tested in groups rather than individually. Although few studies have specifically investigated shoal behaviour in these species, there is evidence of robust social behaviour in the same genera of the species under investigation here. *Coridoras aeneus albinus* is native to South America and is one of the most popular bottom-dwellers in the aquarium hobby. *Corydoras* are highly social benthic catfish that live in slow moving, shallow water and are known for their marked sociality and robust shoaling behaviour in mixed groups of males and females^13,14^.

*Hyphessobrycon megalopterus* is also native to South America and are social fish that live in large mixed groups^15^. A comparative analysis with a shoaling species (*Danio rerio*) showed that groups of fish belonging to *Hyphessobrycon* species were notably more polarized than zebrafish groups^16^, suggesting a tendency to form a school (that is, individuals with a significant degree of synchronization in terms of velocity and alignment) rather than a shoal (a social group with lower cohesion than a school^17^).

*Paracheirodon axelrodi* are small characins living in rivers and flood lagoons of the Amazon basin^18^ that forms large mixed groups. The body of *Paracheirodon axelrodi* is blue/red and is characterized by a contrasting stripe located on the longitudinal direction, a body feature commonly observed in schooling fish. Indeed the presence of such prominent visual cue facilitates the conspecific recognition, mutual orientation and coordination of fish during schooling swimming^19^.

Lastly, *Kryptopterus bichirris* are Asian glass catfish that inhabits large rivers with turbid water in Borneo, Sumatra, the Malay Peninsula^20^. They form large mixed groups and naïve observations in our laboratory suggest a strong degree of cohesion although, as far as we know, no study has assessed whether they form schools.

We expected to observe a general decrease in the preference for the darker area when tested in groups compared to when tested individually, regardless to the body colour of fish. Indeed, swimming in groups has benefits in terms of counter-predator strategies^11–21^ and this may lead social fish to explore bright areas more often than single fish in the same context.

## Results

### Experiment 1: inter-specific comparison (single fish)

**Figure 2:**
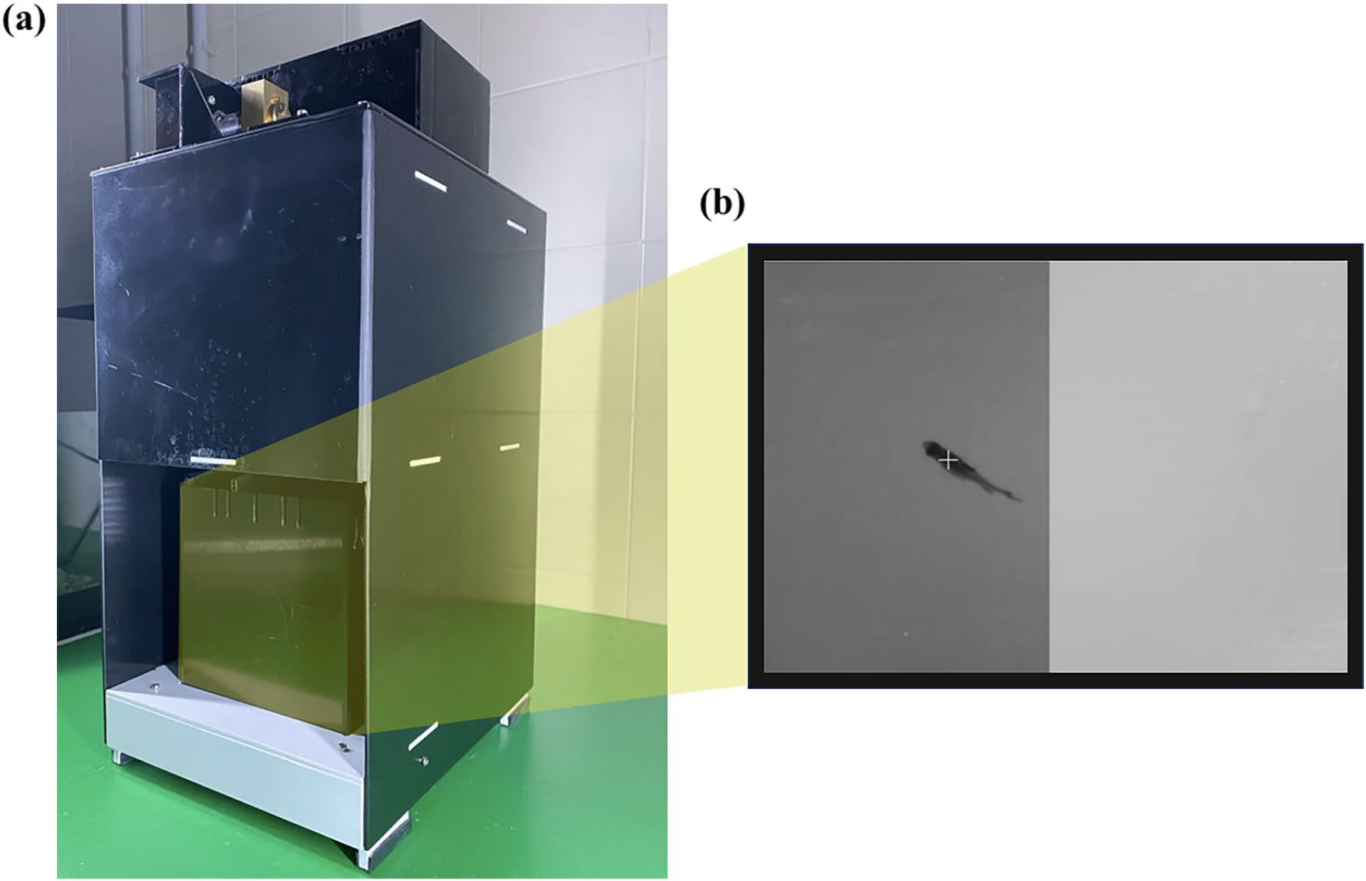
The automated chamber used to record scototaxis. In experiment 1, fish were individually inserted in a chamber (**a**) divided into a light and dark areas. The system automatically records the time spent in the two areas (**b**).

We used the light/dark test to evaluate scototaxis response in fish. Fish were placed in the middle of a non-familiar tank (an automated operant conditioning chamber) whose bottom was transparent. The tank was placed on a screen permitting to split the tank floor into a half-white and half-black area. The screen allowed also to switch the brightness of the two halves of the tank during the experiment (Fig. 2). After five minutes of acclimation in which the screen remained blank, the experiment started (10 min): in the first five minutes (block 1), the black background was displayed on one half of the tank (and the white background on the other), while in the last five minutes (block 2) the position of the black and white backgrounds was switched to avoid any potential side bias. We recorded the proportion of time in the darker area as the dependent variable.

Data were normally distributed (Kolmogorov–Smirnov on the overall proportion of time spent in the darker compartment, all *p*s > 0.200), accordingly, we performed parametrical analyses.

**Figure 3:**
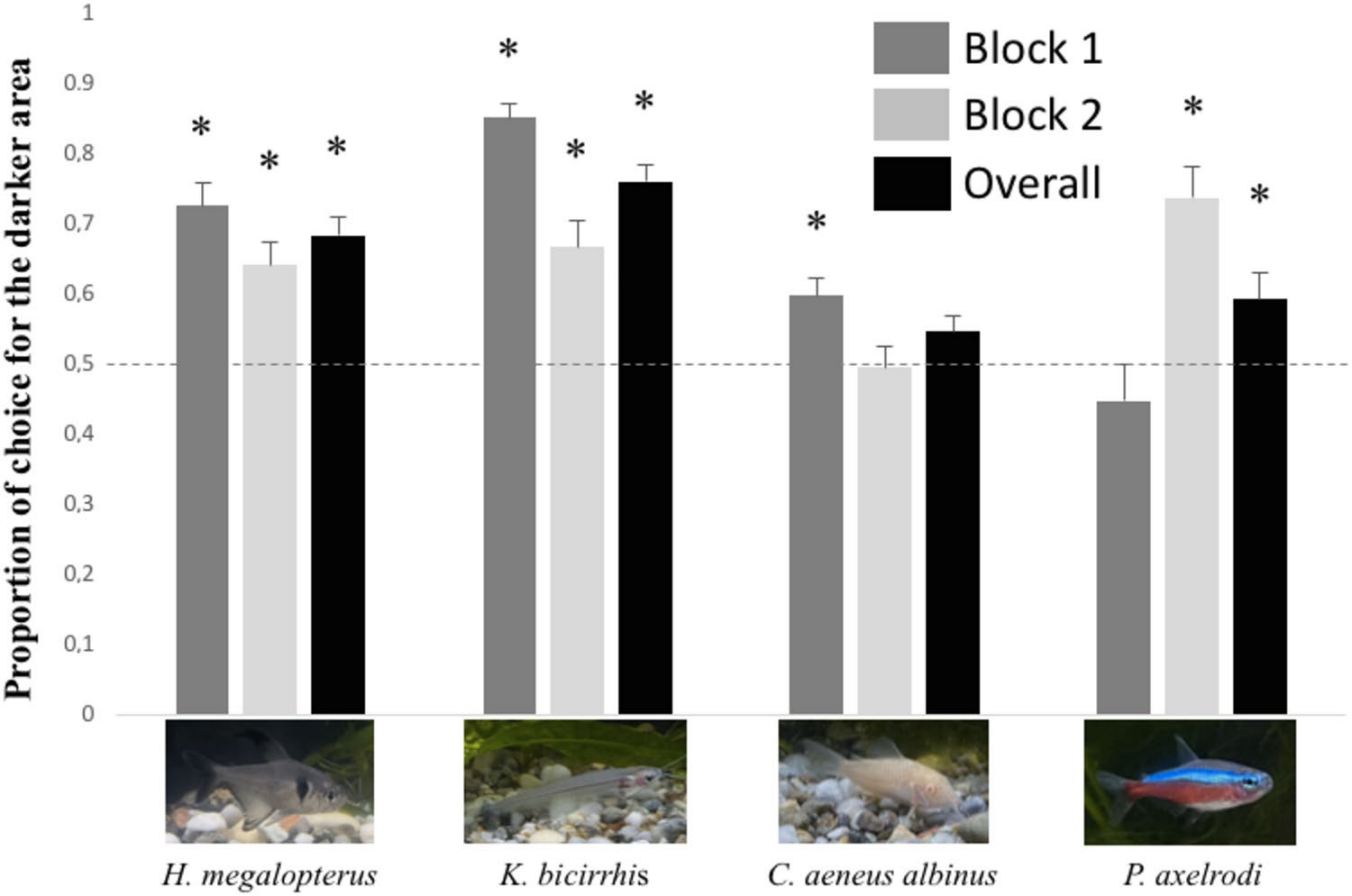
Results of experiment 1. The proportion of time in the darker area is plotted against the four species and the two blocks of the experiment (min 1–5, min 6–10). Asterisks denote a significant departure from chance level (p < 0.05). Bars represent the standard errors.

First, for each species, we analysed whether fish exhibited a significant preference for the darker compartment in the first and second block by performing one-sample t-tests with chance level fixed to 0.5 (Fig. 3). *Hyphessobrycon megalopterus* (dark body colour) spent more time in the darker compartment throughout the whole experiment (block 1, *t*(22) = 6.661, *p* < 0.001, *Cohen’s d* = 1.389; block 2: *t*(22) = 4.335, *p* < 0.001, *d* = 0.904; overall *t*(22) = 7.124, *p* < 0.001, *d* = 1.485). Similarly, *Kryptopterus bicirrhis* (transparent body colour) spent more time in the darker compartment (block 1,* t*(22) = 17.691, *p* < 0.001, *d* = 3.689; block 2: *t*(22) = 4.267, *p* < 0.001, *d* = 0.890; overall *t*(22) = 11.099, *p* < 0.001, *d* = 2.314). *Paracheirodon axelrodi* (iridescent stripe) globally spent more time in the darker compartment but the subjects’ preference emerged only in the second half of the experiment (block 1, *t*(22) = −1.031, *p* = 0.314, *d* = −0.215; block 2: *t*(22) = 5.194, *p* < 0.001, *d* = 1.083; overall *t*(22) = 2.338, *p* = 0.029, *d* = 0.488). On the contrary, *Corydoras aeneus albinus* (bright body colour) spent more time in the darker compartment only in the first half of the experiment but not in the second one (block 1, *t*(22) = 3.762, *p* = 0.001, *d* = 0.785; block 2: *t*(22) = −0.220, *p* = 0.828, *d* = −0.046; overall, *t*(22) = 2.012, *p* = 0.057, *d* = 0.420).

To assess whether the preference for the darker compartment differed as a function of the species and was modulated by the time spent in the apparatus, we performed a repeated measures ANOVA with Species (four species) as between-subjects factor and Block (block 1: min 1–5/block 2: min 6–10) as within-subjects factor. The main effect of Species (*F*(3,88) = 11.235, *p* < 0.001, partial eta squared *η*^*2*^_*p*_ = 0.277) was found revealing a different degree of preference for the darker compartment among the species. Block was not significant (*F*(1,88) = 0.887, *p* = 0.349, *η*^*2*^_*p*_ = 0.010). However, a significant interaction Species × Block was found (*F*(3,88) = 22.837, *p* < 0.001, *η*^*2*^_*p*_ = 0.438) meaning that the tendency to stay in the darker compartment changed over time differently for the species under investigation.

Fisher's least significant difference test (LSD post-hoc analysis) showed a significant difference between *Hyphessobrycon megalopterus* and *Corydoras aeneus albinus* (*p* < 0.001) and between *Hyphessobrycon megalopterus* and *Paracheirodon axelrodi* (*p* = 0.025) while no difference is reported between *Hyphessobrycon megalopterus* and *Kryptopterus bicirrhis* (*p* = 0.062). On the other hand, *Kryptopterus bicirrhis* differed from *Corydoras aeneus albinus* (*p* < 0.001) and *Paracheirodon axelrodi* (*p* < 0.001). No difference between *Corydoras aeneus albinus* and *Paracheirodon axelrodi* was found (*p* = 0.252).

### Experiment 2: inter-specific comparison (shoal)

We used a scaled version of the apparatus, larger in size to insert 20 individuals per species. Again, after five minutes of acclimation fish were recorded a total of 10 min in an apparatus split into a dark and bright compartment. We recorded the average occurrence of the subjects in the white and dark environment at regular intervals of 5 s (120 overall intervals), a dependent variable commonly adopted in behavioural/cognitive studies of fish tested in groups^11,22–24^.

**Figure 4:**
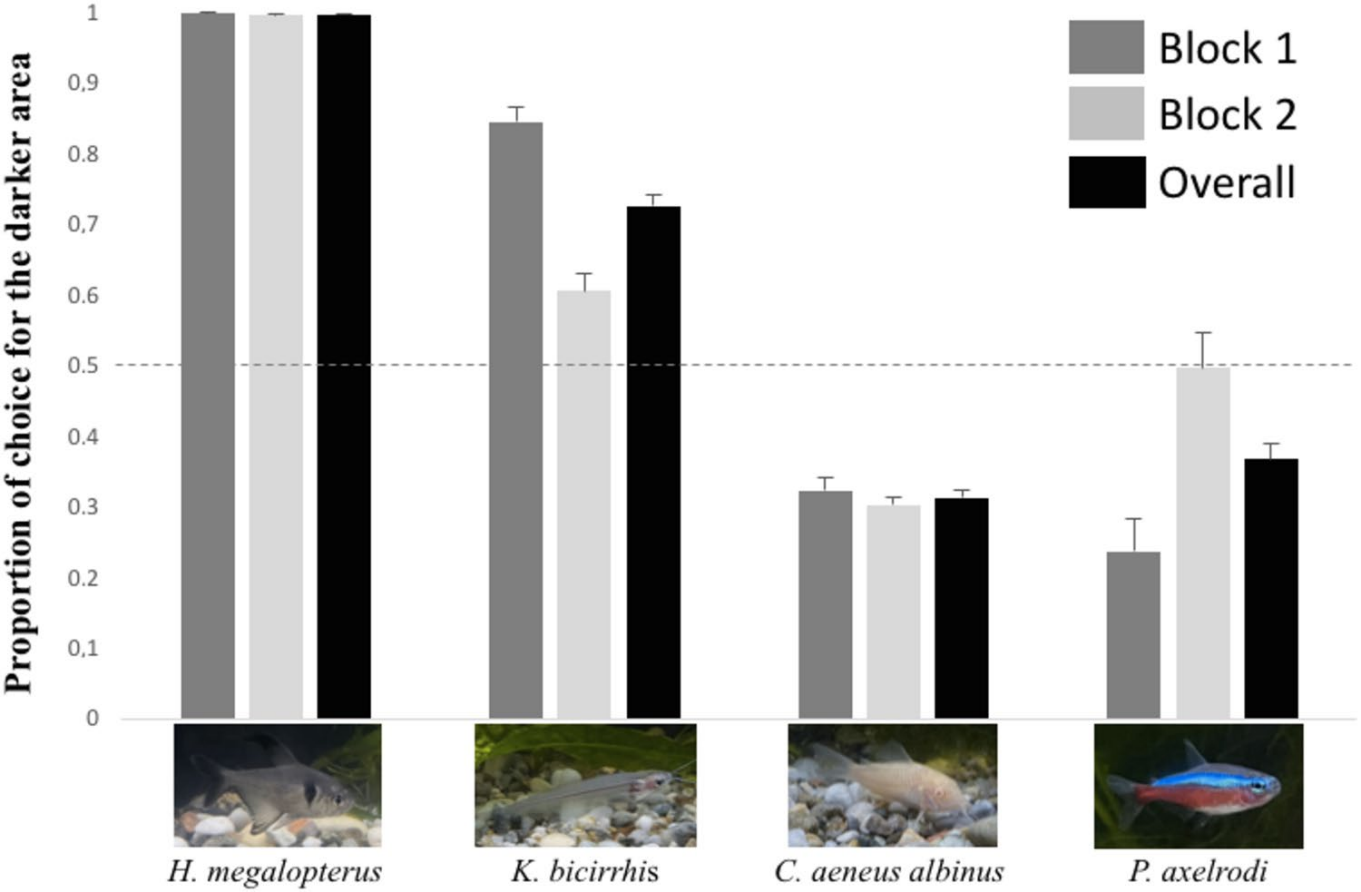
Results of experiment 2. The proportion of individuals in the darker area is plotted against the four species and the two blocks of the experiment (min 1–5, min 6–10). Bars represent the standard errors.

Data (Fig. 4 and Table 1) clearly indicate a consistent choice for the darker compartment for both *Hyphessobrycon megalopterus* (dark body colour) and *Kryptopterus bicirrhis* (transparent body colour). Similarly, a clear preference for the brighter compartment is evident with *Corydoras aeneus albinus* (bright body colour). The case of *Paracheirodon axelrodi* (iridescent stripe) is more ambiguous, with a preference for the brighter compartment that emerges particularly in the first half of the test (Table 1).

**Table 1:** Binomial tests on the frequency of subjects observed in the two halves of the experimental tank (experiment 2).

Species	Block 1 (1–60 intervals) Significant departure from chance	Block 2 (61–120 intervals) Significant departure from chance
*H. megalopterus*	60 times out of 60, p < 0.05	60 times out of 60, p < 0.05
*K. bicirrhis*	45 times out of 60, p < 0.05	19 times out of 60, p < 0.05
*C. aeneus albinus*	21 times out of 60, p < 0.05	28 times out of 60, p < 0.05
*P. axelrodi*	55 times out of 60, p < 0.05	46 times out of 60, p < 0.05

## Discussion

The present study aimed to test whether the spontaneous preference for darker areas exhibited by fish in novel environments is modulated by the colour of the body (experiment 1) and to explore if the presence/absence of other conspecifics in the environment impacts on this scototaxis response (experiment 2). For what concerns the first experiment, the results were in line with most of our expectations. In line with previous literature on other species^1–4^, fish with a dark body colour (*Hyphessobrycon megalopterus*) largely preferred the darker compartment. This tendency is clear for the whole observation time, reinforcing the idea that this is a highly valuable anti-predator strategy in this species. Post-hoc analyses showed that fish with a white-like colour of the body (*Corydoras aeneus aeneus albinus*) had a smaller preference for the darker environment. This preference only occurred in the first half of the test (block 1) and disappeared when analysing the whole observation time. Hiding in dark environments can provide benefits for white-like coloured species too, as only a small amount of light can be reflected by their body and reach the retina of predators. However, we believe that the advantage of staying in a dark environment in the case of *Corydoras aeneus albinus* is counterbalanced by the camouflage with the white floor, thus leading to an overall non-significant choice for either compartment. For what concerns fish with a largely transparent body (*Kryptopterus bichirris*), we were expecting a reduced preference for the darker area compared to *Hyphessobrycon megalopterus* as also in a white background a largely transparent body might partially hide. We did not find this result: they exhibited a consistent preference for swimming in the darker area and they did not display any significant difference from *Hyphessobrycon megalopterus*. Lastly, the species with the iridescent light (*Paracheirodon axelrodi*) represents the most challenging case. In agreement with our expectation, post-hoc analyses showed that their magnitude of choice for the darker area is smaller than *Hyphessobrycon megalopterus*. They spent more time in the darker compartment, although this preference emerged only later, in the second part of the observation time. The observation of their behaviour in our experimental apparatus suggests that this species initially exhibits a thigmotactic behaviour. Also named 'wall hugging', it is an anxiety-like response characterized by the tendency to avoid the centre of an unfamiliar environment and stay close to the boundaries^25,26^. Such behaviour seems to become less frequent across the observation time and in the second part of the experiment fish start to swim preferentially in the darker area. In short, this species might exhibit two alternative behaviours when exploring an unfamiliar environment: a thigmotactic behaviour (although we call for future investigation to directly test it) and a scototactic one.

The second experiment observed the number of fish in a shoal in the bright and dark compartment and suggested important differences in fish behaviour compared to when they are observed one at a time, a fact that—to our knowledge—has been tested only once with zebrafish^12^. *Hyphessobrycon megalopterus* seemed to enhance their tendency to stay in the darker area when swimming in a shoal, as 99% of the fish remained in the darker compartment throughout the whole observation period. Given their dark colouration, it appears particularly adaptive to avoid white backgrounds, in this sense the behaviour of subjects tested in groups might be a more effective anti-predator strategy. The most functional behaviour observed within the shoal may represent another case of the advantage of collective behaviour in the animal kingdom^27,28^. To explain the advantages associated with group choices, two models of collective decision-making have been proposed. The first model is the so-called 'many wrongs' principle^29,30^. According to this model, an individual makes an estimate that is a close approximation to the correct one. If individual errors are randomly distributed around the true mean, they will cancel each other, and the collective performance will be more precise than that of single members. In the case of scototaxis response, ‘errors’ might be represented by a choice for the bright compartment. The second model is called “Meritocratic Leadership”^31^ and applies if some members are more accurate than others to accomplish the task. In our experimental context, the shoal would enjoy an advantage provided that collective decisions are guided by its best-performing members (that is, individuals able to select the darker environment more promptly/consistently). Here there is no possibility to distinguish between the two scenarios, but we cannot exclude that collective behaviour might lead advantages also in *Hyphessobrycon megalopterus*, in particular in the case of the most functional background when exploring an unfamiliar environment. Alternatively, since we used a single trial preference test in which learning is supposed to be minimum or null, we could describe their group behaviour as an example of social facilitation, a phenomenon that occurs when the behaviour of an animal increases the probability of other animals also engaging in that behaviour or increases the intensity of such behaviour^32^.

*Kryptopterus bichirris* confirmed their robust tendency to swim in the darker compartment, showing comparable behaviour both when tested individually and when observed in a group. *Corydoras aeneus albinus*, on the contrary, distributed themselves more often in the bright compartment, unlike experiment 1 where subjects did not show an overall preference but still tended to swim in the darker compartment as soon as placed in the unfamiliar environment. Since the main difference between the two experiments is the absence/presence of other social companions, we can speculate that the tendency of *Corydoras aeneus albinus* to hide in a darker area is social-dependent and may decrease as a function of group size. The possibility exists that, within a shoal, camouflage with the floor is more efficient in the bright area, as each conspecific might contribute with its colour/texture to create a floor-like effect where single individuals can camouflage themselves more efficiently. A single fish in the same white floor may be identified more easily and, hence, *Corydoras aeneus albinus* might prefer a dark area to hide from predators.

Similarly to *Corydoras aeneus albinus*, *Paracheirodon axelrodi* seem to reduce their preference for the darker compartment when observed within a shoal. In the first part of the observation time, they clearly occupied the bright area while, in the second part, they started to exhibit the stigmotactic behaviour described in experiment 1 swimming along the borders and hence staying equally in the two sectors. We may suppose that the iridescent stripe along the body may be poorly visible when a single fish enters a cave, but it may reach a detectability threshold that can be perceived by predators if multiple iridescent stripes are moving in the same cave. In this sense, we might expect that the larger the group of *Paracheirodon axelrodi,* the smaller the preference for the darker areas is.

In this work we generally referred as ‘body colour’ but did not identify the exact perceptual feature (brightness, hue and saturation) underlying fish behaviour nor we distinguished if the performance is based on the absolute colour of the species or the contrast between body colour and the background. Also, we are aware that, in the attempt to test the role of body colour/contrast with the background, we selected different species, an approach commonly used in studies on body colours of animals^33–35^. However, this comparative approach cannot exclude that the differences among these species are a result of selection on other traits rather than colour. We selected species belonging to two different order: characiformes (*Paracheirodon axelrodi* and *Hyphessobrycon megalopterus*) and siluroformes (*Corydoras aeneus albinus* and *Kryptopterus bichirris*). The two orders diverged approximately 90–100 million years ago^36^ and it is easy to expect inter-specific differences other than body colour. That said, even though the four species share some colours, they are also clear representative examples of the four chromatic categories under investigation. We could not test the hypotheses of these colour/contrast with the background within the same species or closely related ones in the attempt to limit inter-species differences. We call for future studies to extend the investigation to more species within these four categories (darker, brighter, transparent and iridescent body) to confirm whether the differences found here are the result of colour/contrast only.

Another important limitation of the study is the difficulty to compare the results of the two experiments. Since automatic tracking of the fish was not possible with multiple fish hiding in the dark (Experiment 2), we needed to record a different dependent variable, the proportion of fish found in the darker area at constant intervals (rather than the proportion of time of single fish in the darker compartment of the tank of experiment 1). This prevented us from making a direct (statistical) comparison between the two experiments. Also, the apparatus of Experiment 2 was not a mere scaled version of that used in Experiment 1 but differed with respect to some methodological details. For instance, the different water level was necessary to include more subjects in the tank without any significant stress for the animal and to control for population density. However, we cannot exclude that this different level of water between the two experiments might have influenced the exploratory behaviour of our subjects. That said, we believe that fish preferences for the two sectors in Experiment 2 were unambiguous for at least three species (*Hyphessobrycon megalopterus* and *Kryptopterus bichirris*, and *Corydoras aeneus albinus*) regardless of the dependent variable adopted. Our study clearly points toward the idea that scototaxis response of fish (and presumably other animals) is far from being universal but context-dependent and is influenced by the colour/contrast of the species and, presumably, by the presence/absence of other conspecifics in the same environment. Species that can also (largely or partially) hide in a bright environment may still exhibit a preference for the darker area, although to a lesser degree. In addition, fish may reverse their preference when swimming in groups, probably because the advantages of entering dark areas are counter-balanced by other advantages derived by hiding within a shoal. As most fish species are social and swim in shoals, the behaviour observed here in the social context may indeed reflect the behaviour exhibited by these species in their natural environment.

## Methods

### Experiment 1: inter-specific comparison (single fish)

#### Subjects

A total of 96 freshwater fish were tested, 24 for each species (*Corydoras aeneus albinus, Paracheirodon axelrodi, Kryptopterus bicirrhis*, and *Hyphessobrycon megalopterus*). Subjects were adult sexually mature individuals (half males and half females) maintained in groups of 26 (contained in 150-L tanks) at the Laboratory of Comparative Psychology of the University of Padova. An 18-W fluorescent light was provided above each stock tank (photoperiod was 14:10 h light:dark), each tank also has air filters, natural gravel, and live plants. The water temperature was set to 26 ± 1 °C. Fish were fed twice daily with commercial food flakes in the morning and with live brine shrimp (*Artemia salina*) in the afternoon.

#### Experimental apparatus

We used a “Zantiks AD” (Fig. 2) chamber, an automated operant conditioning chamber recently used to test cognitive skills in zebrafish^7,37,38^. The overall unit size includes a computer, software, and the experimental tank (width × length × height: 22 × 30 × 50 cm). The experimental tank (14 × 20 × 15 cm) was made up of black plastic walls and a transparent plastic floor. It was filled with 7 cm of water. The device is connected to a wireless router allowing us to operate it with our laptop. Subjects’ position was automatically detected by the integrated computer through an infrared camera (placed above the tank) and an infrared source (positioned underneath the tank).

#### Procedure

Subjects were gently collected from the stock tank by a fish net and singly placed in the center of the experimental tank. After five min of acclimation, in which the bottom of the tank was black, half of the floor became white (RGB: 255, 255, 255) while the other half of the floor remained black (RGB: 0, 0, 0). Five minutes later, the position of the light and dark floors was switched to ensure that fish choice was not influenced by any existing laterality bias in the environment^20^. As dependent variables, we recorded the proportion of time spent in the dark compartment. To ensure that fish have noticed the differences in the background, fish were included in the analyses only if they explored both sectors at least once during the 10-min observation. This occurred 100% of the cases.

### Experiment 2: inter-specific comparison (shoal)

#### Subjects

A total of 80 fish were tested, 20 for each species (half males and half females). Fish were maintained in the same conditions and stock tanks described in Experiment 1.

#### Experimental apparatus

Like most of the apparata used in scototaxis tests^8,9,39,40^, this was not an automated chamber. The apparatus (40 × 60 × 40 cm) was similar to the one described in Experiment 1. The size was adapted to host 20 individuals during the observation time. The volume was indeed 20 time larger than the volume of the experimental chamber used in the experiment 1, to keep constant population density (1:20). To create a dark and white environment, black and white plastic material (poliplack^©^) covered both the four walls and the floor. Half of the tank was covered by white material, while the other half was covered by black one. In addition, a lamp (18 W) was placed in correspondence with the white compartment to brighten the white area. The tank was filled with 16 cm of water.

#### Procedure

Subjects of each species were inserted together in the middle of the experimental tank. After five min of acclimation, we video-recorded fish behaviour for 10 min with a camera placed one meter above the experiment tank. After each test, the tank was rotated by 180° to eliminate any potential spatial bias. Videos were analysed by two independent blind observers. Every five seconds they counted the number of fish in the bright and dark compartments (a total of 120 measures for each video). Inter-rater reliability correlation was equal to 1.

### Ethics declarations

The study complies with all laws of the country in which it was performed (Italy) and was approved by the local ethics committee of the University of Padova (12/2021). The authors complied with the ARRIVE guidelines.

## Data Availability

The datasets generated during the current study are available here: https://osf.io/sj8cv/.
